# The Mediating Role of Insomnia and Exhaustion in the Relationship between Secondary Traumatic Stress and Mental Health Complaints among Frontline Medical Staff during the COVID-19 Pandemic

**DOI:** 10.3390/bs10110164

**Published:** 2020-10-26

**Authors:** Ica Secosan, Delia Virga, Zorin Petrisor Crainiceanu, Tiberiu Bratu

**Affiliations:** 1 Faculty of Medicine, Victor Babes University of Medicine and Pharmacy, 300041 Timisoara, Romania; secosan.ica@umft.ro (I.S.); zcrainiceanu@gmail.com (Z.P.C.); office@brol.ro (T.B.); 2 Department of Psychology, West University of Timisoara, 325100 Timisoara, Romania

**Keywords:** insomnia, frontline clinicians, exhaustion, stress, mental health, SARS COV-2

## Abstract

The outbreak of coronavirus disease (COVID-19) brought significant psychological implications for healthcare professionals. We aimed to investigate the serial mediation effect of insomnia and exhaustion in the relationship between secondary traumatic stress (STS) and mental health complaints among the frontline healthcare professionals during the COVID-19 pandemic. In this cross-sectional study, 126 frontline healthcare workers from Romania completed validated surveys between March and April 2020. PROCESS macros were used to test the proposed hypotheses of the three-path mediation model. We computed the models for insomnia as the first mediator (M1) and exhaustion (M2) as our second mediator. STS was significantly related to insomnia. Insomnia was significantly related to exhaustion, and STS was positively related to exhaustion. In the third model, exhaustion was strongly and positively related to mental health complaints. The total indirect effect was positive, and the sequential indirect impact of STS on mental health complaints via both mediators in series (insomnia and exhaustion) was significant. Secondary traumatic stress had a positive direct effect on mental health complaints. In our limited sample, the results show that frontline medical staff during the COVID-19 outbreak have high STS, which are related to mental health complaints through insomnia and exhaustion.

## 1. Introduction

The COVID-19 (coronavirus disease) is an infectious disease caused by a newly discovered coronavirus that spreads primarily through droplets of saliva and discharges from the nose when an infected person coughs or sneezes [1]. The outbreak of COVID-19 caused considerable public concern worldwide and brought substantial psychological implications for the general population and particularly the medical staff. By July, 3335 medical professionals were tested positive for COVID-19 in Romania. Among the infected medical team, there were physicians, auxiliary medical personnel, nurses, and caregivers. Worldwide, thousands and maybe more doctors and medical staff are already infected with the new coronavirus.

Increasing patient volume, the overwhelmed hospitals, lack of specific drugs, uncertainty, and fear for their safety may all contribute to healthcare workers’ psychological distress. Previous studies on SARS (severe acute respiratory syndrome) and Ebola have reported a high level of emotional distress during the outbreaks of such epidemics [2]. Studies have demonstrated that 18%–57% of medical staff experienced emotional distress during and after the outbreak of the infection [3]. Clinicians on the frontline showed a higher intrusion sub-score than other healthcare professionals during the 2015 MERS (Middle East respiratory syndrome) outbreak [4].

As past studies already showed, the psychological distress during a pandemic may be higher and more sustained among frontline healthcare workers [5]. Stress, alongside other psychological implications for health professionals during a pandemic, is considered the cause of insomnia. Much research has been published regarding that issue during the previous SARS outbreak. A longitudinal study performed in 2003 identified low quality of sleep in nurses caring for SARS patients even one month after caring for SARS patients and one month after the hospital resumed normal operations [6]. Nowadays, as one study shows, more than one-third of the medical staff suffered from insomnia symptoms during the COVID-19 outbreak [7].

The medical population working on the frontline may display a high prevalence of stress symptoms, such as emotional exhaustion and burnout that, in turn, may also predispose the clinicians to a variety of mental health problems. The burnout syndrome is defined as a, “prolonged response to chronic stressors on the job”, which involves emotional exhaustion, depersonalization, and decreased individual accomplishment [8]. As many studies showed, physicians have a higher prevalence of burnout than the general population and any other profession. A past study investigated the associations between individual and job characteristics, sleep disturbances, and mental health conditions among firefighters and reported that psychological distress was significantly associated with sleep disorders [9].

Moreover, healthcare workers are at high risk of indirectly exposure to trauma through their work and may suffer from symptoms of secondary traumatic stress (STS). The secondary traumatization is related to the professionals’ indirect exposure to trauma through the patient’s narratives and accounts of the traumatic events. This can happen when assisting the patient recovering after such an event, which involves listening to details about the traumatic event [10].

To date, the effect of STS on other work-related symptoms, such as mental health complaints, has been tested, but few studies have addressed the potential mechanism underlying the relationship between STS and mental health complaints, that is still not clear, but mediators may be involved. Prior research has concentrated primarily on the relationship between STS and other variables, such as burnout, mental health complaints, and insomnia. There has been less consideration of the role of insomnia and exhaustion in mediating the relationship between STS and mental health complaints.

Previous studies have consistently found that mediation analysis is employed to explore the underlying mechanism or process by which one variable influences another variable through a mediator variable [11], rather than a direct causal relationship between the independent and dependent variable. For example, as one study showed, the worker burnout plays a mediating role between the impact of external job demands and work-related outcomes [12]. Also, based on the job-demands-control model, exhaustion fully mediates the relationship between job demands and mental health [13]. Sleep disorders, such as insomnia, were studied as a predictor of stress-related symptoms in various studies, confirming that sleep problems play a mediating role in the relationship between occupational stress and physical health problems. Thus, exploring the underlying mechanism by which STS is related to mental health complaints, mediated by insomnia and exhaustion, serves to clarify the nature of the relationship and understand the impact of the studied variables.

A high level of work-associated stress characterizes the ICU (Intensive Care Unit) and Emergency Medical Services due to emotional and physical pressure, which are factors known to increase the risk of burnout and STS. Exposure to severe and chronic stressors generates work-related stress symptoms, such as burnout and STS, that, in turn, may also predispose medical professionals to a variety of mental health problems. Before COVID-19, numerous studies showed that sleep disorders and mental health complaints are associated with burnout [14]. Furthermore, as one prospective multi-sited study showed, an essential percentage of ICU healthcare workers presented a high burnout [15]. A previous study conducted in 2019 on the same hospital, County Emergency Hospital Pius Brinzeu Timisoara, Romania, among Emergency Services using multiple linear regression analysis, emphasized that healthcare staff mental health is affected by burnout, insomnia severity, and STS. At that time, we found that mental health was predicted by exhaustion, insomnia severity, and secondary traumatic stress [16].

Our study brings mediators’ unique perspective in the relationship between STS and mental health complaints. In our opinion, it is of most importance to understand the relationship between different types of psychological implications on frontline medical staff’s work-experience during a pandemic time. Therefore, we believe that our model could be a first step in identifying strategies to prevent and treat these issues in order to ensure the frontline healthcare workers’ well-being, professional satisfaction, and improved work environments, during a very stressful time, the COVID-19 pandemic.

Secondary traumatic stress occurs due to healthcare workers’ exposure to suffering patients by internalizing some of the emotional energy from each interaction. Thus, exposure to suffering is the first pathway to STS [17]. When negative energy accumulates without counteraction by some positive mechanisms, this residual energy wreaks within the affected person [18]. Numerous studies showed that psychological trauma has strong associations with poor mental health [19]. Moreover, traumatic stress exposure is linked to increased risks and severe acute and chronic illnesses [20]. As one study demonstrated, some of those symptoms involve insomnia and sleep disturbances [21]. This is consistent with other studies that cited that those symptoms led to dissatisfaction at work, lowered organizational commitment, and exhaustion in addition to high levels of absenteeism [22,23]. Considering those described above, we can argue that exposure to trauma, which leads to STS, creates a pathway to other symptoms, such as insomnia, feelings of detachment, exhaustion, with all of those having a severe impact on healthcare employee mental health.

Also, the secondary traumatic stress is seen as an occupational risk factor [24] with an empathic nature through which others’ experiences are transformed into personal. It is felt as if the background is an individual circumstance [25]. Therefore, since those are symptoms similar to the primary trauma [26], it increases the risk of several psychological symptoms, such as anxiety, sleep disturbances, depression, fatigue, and exhaustion [25,27]. Moreover, as previous studies showed, STS was included in a salient job demand model [28,29]. Following a well-researched Job Demands-Resources model (JD-R), two critical forces affect burnout: Job demands and job resources. Many studies already showed a close relationship between STS and burnout [30,31]. Being understood as job demand, STS was related more closely with exhaustion and depersonalization, two of the sub-constructs of burnout [19]. One study showed that the participants try to cope with high levels of STS by emotionally detaching themselves from the exhaustion, which follows the symptoms of STS [25].

In conclusion, STS is a significant threat to mental health through insomnia and exhaustion, which should be dealt with proactively to mitigate their effect on medical staff health. Therefore, in our opinion, research must focus not just on the psychological outcomes of the COVID-19 pandemic, but also on the mediators of the relationship between work-related stress symptoms and other outcomes, such as STS and mental health complaints among frontline healthcare workers. One possible mediator is insomnia, a short-term or long-term disorder, with trouble falling and/or staying asleep. Several studies conducted from January until March 2020 have reported that doctors and nurses working on the frontline experienced anxiety, depression, and sleep problems [32]. Another possible mediator is exhaustion, as a part of burnout, which refers to feelings of depletion and feeling down, resulting from overtaxing work [33]. It is experienced by a wide range of employees, including healthcare workers [34]. Furthermore, the effects of exhaustion have an impact on frontline medical workers by experiencing mental health complaints.

In the present study, we hypothesized and tested a three-path mediation model, considering insomnia and exhaustion as mediators that link STS and mental health outcomes, therefore highlighting the focus on secondary traumatization and mental health complaints, via their mediators, insomnia and exhaustion, among frontline workers during the COVID-19 pandemic. 

We believe that this study may contribute to understanding the relationship between STS and mental health complaints from the standpoint of its mediators, insomnia, and exhaustion. 

**Figure 1:**
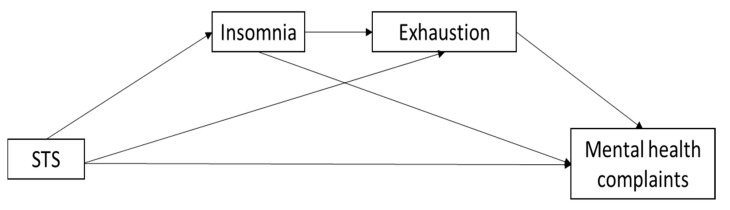
Hypothetical model.

In summary, we hypothesize that frontline workers who suffer from STS are more likely to experience insomnia. Insomnia will be positively related to exhaustion, which, in turn, will positively relate to mental health complaints. Finally, STS indirectly and positively relates to mental health complaints through the serial mediating influence of insomnia and, in turn, exhaustion. Figure 1 summarizes the hypothesized model:

**Hypothesis** **1** **(H1).** *STS is positively related to insomnia.*

**Hypothesis** **2** **(H2).** *Insomnia is positively related to exhaustion.*

**Hypothesis** **3** **(H3).** *Exhaustion is positively related to mental health complaints.*

**Hypothesis** **4** **(H4).** *STS is positively and indirectly related to mental health complaints, mediated by insomnia and exhaustion.*

## 2. Material and Methods

### 2.1. Study Design

The present study is a cross-sectional one; all data were collected from March to April 2020 in County Emergency Hospital Pius Brinzeu Timisoara, Romania, among Emergency Services.

### 2.2. Participants and Procedure

We have surveyed frontline healthcare workers, emergency doctors, ICU doctors, and medical nurses from two Hospital Departments (Emergency and ICU) in Romania, namely the County Emergency Clinical Hospital Pius Brinzeu, Timisoara. The inclusion criteria concerned the categories of personnel who directly contact patients during the COVID-19 outbreak, who directly contact patients during the COVID-19 outbreak through the performed medical act, encompassing primary doctors, specialists, residents (trainees), ICU, and emergency medicine nurses. Other categories of staff and auxiliary personnel were excluded from the research. All data were collected online via a link sent by email. According to the hospital classification system of The Romanian Ministry of Health, all hospitals in Romania are classified into county emergency hospitals, city hospitals, and specialized hospitals. A county emergency hospital must have a bed number exceeding 500 and provides comprehensive and specialized medical care with a high level of medical education and research functions. Our cluster sampling procedure was used to recruit 200 frontline healthcare workers from The County Emergency Hospital Pius Brinzeu Timisoara, Romania, affiliated hospital of the Victor Babes University of Medicine and Pharmacy Timisoara, Romania. Each of the participants was provided with an online questionnaire, and 126 of them returned the survey (response rate = 63%). There were no missing data or invalid responses. 

Following the Romanian Ministry of Health Order number 533/03.29.2020 regarding the Plan of measures for hospitals’ preparation in the COVID-19 pandemic, County Emergency Hospital Pius Brinzeu Timisoara, Romania took over the critical cases of patients infected with the novel coronavirus. The usual hospital activity was decreased by 80% regarding chronic cases to increase the hospital’s resources in treating COVID-19 patients. By May 2020, there were 19,133 COVID-19 patients in Romania, 98,403 people in isolation, and 2993 people in official quarantine. Although Timis County had, by the end of May, 505 confirmed cases since the outbreak of the novel coronavirus crisis in Romania in early March, the increase in demand and changes to supply, the reorganization of hospital facilities, redeployment of staff, extended work tasks, increase in donning and doffing PPE, and implementing new guidelines and protocols, caused tremendous psychological pressure for the frontline healthcare workers.

### 2.3. Study Variables and Data Collection

The Secondary Traumatic Stress Scale is a self-report inventory designed to assess the frequency of STS symptoms in professional caregivers. Respondents indicate how often they experienced each of the 17 STS symptoms in the past seven days (ranging from 1 “never” to 5, “very often”). The items are organized in three subscales: Intrusion (5 items, e.g., “My heart started pounding when I thought about my work with clients”), avoidance (7 items, e.g., “I was less active than usual.”), and arousal (5 items, e.g., “I expected something bad to happen.”). A total score below 28 corresponds to “little or no STS,” a score between 28 and 37 means “mild STS,” between 38 and 43 “moderate STS,” between 44 and 48 “high STS,” and beyond 49 “severe STS” [35].

Mental Health Complaints were measured with the MHI-5 screening test [16]. This scale comprises five items, evaluated on a 6-point Likert scale, ranging from 1 = never, and 6 = always (e.g., “During the past month, how much of the time have you felt calm and peaceful?”). Items 2 and 4 were recorded. A high score indicated poor mental health [36].

The Insomnia Severity Index is composed of seven items (e.g., “How worried/distressed are you about your current sleep problem?”), rated on a five-point Likert scale (0—not at all, 4—extremely), and the time interval is ‘in the last two weeks’. Total scores range from 0 to 28, with high scores indicating greater insomnia severity (0–7 = no clinically significant insomnia; 8–14 = subthreshold insomnia; 15–21 = clinical insomnia; 22–28 = clinical insomnia/severe) [37].

The exhaustion dimension was evaluated based on the Maslach Burnout Inventory MBI-GS, an instrument designed to assess the three components of burnout syndrome: Emotional exhaustion (EE), depersonalization (DP), and reduced personal accomplishment (PA). Exhaustion has 5 items (e.g., “I feel emotionally drained from my work.”). Respondents were asked to evaluate the items on a seven-point scale from 0 (never) to 6 (always). The items are answered in terms of the frequency with which the respondent experiences these feelings, on a 7-point, fully anchored scale (ranging from 0—”never” to 6—”always”) [38].

### 2.4. Data Analysis

The correlation between the study variables (insomnia, secondary traumatic stress, exhaustion, mental health complaints) was examined using Pearson correlation coefficients. Correlational analysis for the four variables was performed using the Statistical Package for Social Science (SPSS) v21 program (IBM Corp., Armonk, NY, USA). The significance level adopted was *p* ≤ 0.05. Two-tailed correlations between all the variables were calculated. We conducted a path analysis to test the mediation effects. Mediation analysis is used to identify and explain the relationship between the dependent variable Y and an independent variable X, which may be affected via a third variable W. W is a mediating variable, and it represents a mechanism through which X affects Y [39]. In the current study, STS impacts insomnia and exhaustion, with “insomnia” and “exhaustion” acting as mediator variables, which further affects the mental health complaints.

Finally, we have used PROCESS macro using the SPSS v21 program to test our mediation hypothesis using a bootstrapping procedure by Hayes [40], using one independent variable (STS), two mediators (insomnia, exhaustion), and one dependent variable (mental health complaints). We calculated 95% confidence intervals (CIs) based on bias-corrected bootstrap analyses with 5000 repetitions to analyze indirect effects.

### 2.5. Ethical Aspects

All gathered information was confidential; the participation was entirely voluntary and written informed consent was obtained from all the participants. The Ethics Committee approved this study of the County Emergency Clinical Hospital (No. 170/05.08.2019) as part of ongoing research considering the burnout syndrome and psychological implications on the healthcare profession.

## 3. Results

A total of 126 health professionals took part in the survey: 32 nurses and 94 physicians were questioned. Socio-demographic data were collected on gender (male or female), marital status (single, married, divorced, widowed), parental status (children; yes or no), profession (physician or nurse), technical title (trainee, specialist, primary or other), and specialty (ICU or emergency medicine specialist). 

The demographic characteristics of participants are summarized in Table 1. During the study period, the County Emergency Clinical Hospital Pius Brinzeu, Timisoara, was actively involved in the care of COVID-19 patients. 

Table 2 shows the correlation analysis, descriptive statistics, and scale reliabilities. Thus, STS correlated positively and significantly with insomnia (*r* = 0.59, *p* < 0.001) and positively and significantly with exhaustion (*r* = 0.47, *p* < 0.001), and with mental health complaints (*r* = 0.38, *p* < 0.001). Insomnia was negatively and significantly related to exhaustion, and mental health complaints (*r* = 0.39, respectively *r* = 0.27, *p* < 0.001). Finally, exhaustion was positively and significantly related to mental health complaints (*r* = 0.56, *p* < 0.001). Overall, we observe strong positive correlations among all variables of the model.

As depicted in Table 3, we first computed the model for insomnia as the first mediator (M1). STS was significantly related to insomnia (b = 0.39, *p* < 0.001), and this result offer support for Hypothesis 1. A high level of insomnia predicts high secondary traumatic stress. Next, we computed the analyses for exhaustion (M2) as our second mediator. Insomnia was significantly related to exhaustion (b = 0.25, *p* < 0.01) supporting Hypothesis 2. In this second model, also STS was positively related to exhaustion (b = 0.28, *p* < 0.001). In line with Hypothesis 3, in the third model, exhaustion was strongly and positively related to mental health complaints (b = 0.31, *p* < 0.001).

Consistent with Hypothesis 4, the total indirect effect was positive, and the sequential indirect effect of STS on mental health complaints via both mediators in series (insomnia and exhaustion) was significant (b = 0.03, 95% CI [0.000, 0.084]). Based on these results, we can conclude that sequential mediation is statistically significant. STS had a positive direct effect on mental health complaints (b = 0.15, 95% CI [0.076, 0.230]. Interestingly, there was also a positive indirect effect of STS on mental health complaints through insomnia (b = 0.06, 95% CI [0.015, 0.122]) and exhaustion (b = 0.08, 95% CI [0.034, 0.142]). Overall, there is partial mediation support for the proposed theoretical model. Therefore, the relationship between insomnia and mental health complaints is partially mediated by secondary traumatic stress and exhaustion.

## 4. Conclusions and Discussion

The purpose of this research was to study the serial mediation effect of insomnia and exhaustion in the relationship between secondary traumatic stress and mental health complaints among frontline healthcare workers during the COVID-19 pandemic.

Firstly, we found that STS is positively related to insomnia. Frontline healthcare workers who experience isolation, anxiety, dissociation, physical ailments, and helplessness are more likely to develop sleeping problems. Those results are in line with other studies, which demonstrated that sleep and other mental health problems are associated with an increased risk of work related-stress symptoms. In contrast, insomnia and sleep deprivation during overnight work mediate these relationships.

Secondly, we observed a positive relationship between insomnia and exhaustion. The frontline medical staff is under enormous pressure during the COVID-19 pandemic. They face higher expectations, leading to anxiety, depression, stress-related symptoms, insomnia, and worry about becoming infected or infecting their family members. All those symptoms can lead to exhaustion. We already know from previous studies that stress is considered the primary cause of insomnia. Our results align with past research that identified a relationship between insomnia and other related psychological effects of working in hospitals during the previous SARS outbreak [41].

Thirdly, our study showed that exhaustion is positively related to mental health complaints. Research in this field showed that increased burnout levels are associated with higher mood disturbance and lower general and mental health. Our results are sustained by other studies in this field, who suggested that among the work-related stress symptoms associated with mental health complaints, being emotionally exhausted at work plays an essential role [42].

Finally, our hypothesis was confirmed, as STS is positively and indirectly related to mental health complaints, mediated by insomnia and exhaustion. In our limited sample, we found that clinicians who presented with higher STS levels are more likely to have sleep problems, such as insomnia. Therefore, they experience exhaustion at work, which increases their mental health complaints.

This research could be considered an initial attempt to integrate the three paths mediational hypothesis, which, to our understanding, is new and unique in Romanian frontline healthcare workers during the SARS COV-2 pandemic. 

### Weaknesses and Suggestions for Further Research

The results of this study should be evaluated, considering several limitations. One of the limits is the cross-sectional design. Our research cannot assess if there will be a change in variables over time. The relations found do not involve causal inferences between the studied variables. Regarding causality, we cannot be sure that STS causes insomnia and exhaustion and/or that STS causes health complaints. Moreover, the sample size was too small to assume that our predictive model fits all frontline healthcare workers’ work-experience during the COVID-19 pandemic in Romania. Further research with a larger sample of participants, such as a nation-wide study, should be performed to obtain a complete picture of all the short- and long-term health consequences of healthcare workers exposed to the COVID-19 crisis.

Regarding other potential confounds, the majority of studies investigate the socio-demographic correlates of burnout. Still, as one systematic review showed, results are not consistent and offer little support to those variables [43]. On the other hand, due to a multivariate analysis among ICU healthcare workers, considering different variables, only gender was identified as a risk factor [15]. Working hours per week, several night shifts per month, lack of compensation, and other professional factors were not correlated to burnout among intensivists before the COVID-19 outbreak. However, future investigation of those factors may clarify the impact of possible confounds on healthcare staff mental health. Longitudinal studies could further strengthen our conclusions and evidence of the nature of the relationships between STS and mental health complaints. Future research should also test other mediators in relation to mental health complaints to provide a broader image of the psychological implications in treating patients on the frontline during the COVID-19 outbreak.

In conclusion, these findings suggest that psychological manifestations such as secondary traumatic stress, mental health complaints, insomnia, and exhaustion should be considered when investigating the COVID-19 exposure of frontline healthcare workers during the pandemic crisis. Our findings can be used to formulate psychological interventions focused not just on the psychological outcomes of the COVID-19 pandemic, but also on the mediators of the relationship between work-related stress symptoms and mental health outcomes, such as insomnia and exhaustion. Current and future studies among frontline healthcare workers during the COVID-19 medical crisis may provide valuable information for additional training and organizational support to improve frontline healthcare workers’ mental health and psychological resilience during the COVID-19 pandemic.

## Figures and Tables

**Table 1:** Demographic and professional characteristics of frontline healthcare workers.

Variables	Categories	Frequency	Percents
Gender	Male	45	35.7
	Female	81	64.3
	Total	126	100.0
Marital status	Single	54	42.8
	Married	66	52.3
	Divorced	6	4.7
	Widower	0	0.0
	Total	126	100.0
Children	Yes	70	55.5
	No	56	44.4
	Total	126	100.0
Profession	Physician	94	74.6
	Nurse	32	25.3
	Total	126	100.0
Staff category-doctors	Trainee	57	45.2
	Specialist	19	15.0
	Primary	21	16.6
	Other	29	23.0
	Total	126	100.0
Specialty	ICU	46	36.5
	EM	80	63.4
	Total	126	100.0

**Table 2:** Descriptive statistics, correlations, and scale reliabilities.

Variable	M	SD	1	2	3	4
1. Insomnia	10.56	2.84	(0.91)			
2. STS	30.27	3.58	0.59 **	(0.94)		
3. Exhaustion	46.26	6.65	0.39 **	0.47 **	(0.92)	
4. Mental health complaints	44.36	10.08	0.27 **	0.38 **	0.56 **	(0.91)

Note: *n* = 126, reliability coefficients are reported along the diagonal. ** *p* < 0.001 (two-tailed).

**Table 3:** Total, direct, and indirect effects of the mediation model (PROCESS).

Variables	Unst. Coeff.	SE	*p*	BC Bootstrap 95% CI	
				Lower	Upper
The total effect of					
STS->MHC	0.34	0.02	0.000	0.29	0.39
The direct effect of:					
STS->MHC	0.15	0.03	0.00	0.07	0.23
STS->In	0.39	0.02	0.000	0.34	0.44
STS->Ex	0.28	0.05	0.000	0.18	0.38
In->MHC	0.17	0.07	0.01	0.03	0.31
In->Ex	0.25	0.10	0.01	0.05	0.45
Ex->MHC	0.31	0.06	0.000	0.19	0.43
The indirect effect of:					
STS->In->MHC	0.06	0.02		0.01	0.12
STS->Ex->MHC	0.08	0.02		0.03	0.14
STS->In->Ex->MHC	0.03	0.02		0.00	0.08

**Note.***n* = 126; Number of bootstrap samples for percentile bootstrap confidence intervals: 10000. In = Insomnia; STS = Secondary traumatic stress; Ex = Exhaustion; MHC = Mental health complaints.

## Abbreviations

COVID-19	coronavirus disease
SARS	severe acute respiratory syndrome
MERS	Middle East respiratory syndrome
JD-R	Job-Demands-Resources Model
EE	emotional exhaustion
DP	depersonalization
PA	reduced personal accomplishment
SPSS	Statistical Package for Social Science
CIs	confidence intervals
In	insomnia
MHC	mental health complaints
